# Understanding accounting professionals’ intention to adopt robotic process automation: a TOE-based empirical assessment from an emerging country

**DOI:** 10.3389/frobt.2025.1747539

**Published:** 2026-01-29

**Authors:** Nusirat Ojuolape Gold, Husain Coovadia, Katlego Thipe

**Affiliations:** 1 Department of Commercial Accounting, College of Business and economics, University of Johannesburg, Johannesburg, South Africa; 2 Department of Accounting and Finance, Faculty of Management and Social sciences, Kwara State University, Malete, Nigeria

**Keywords:** accounting professionals, behavioral intention, developing country, emerging technologies, robotic process automation, TOE framework

## Abstract

The proliferation of the Fourth Industrial Revolution (4IR) is transforming the accounting landscape, with technologies such as Robotic Process Automation (RPA) changing the face of traditional accounting processes. This study investigates the level of RPA adoption among accountants in South Africa and examines how technological–organizational–environmental (TOE) factors influence the behavioral intention of RPA adoption. The study employed an exploratory cross-sectional survey comprising responses from 100 professional accountants in practice to analyze its data, combining descriptive statistics with a multiple linear regression model supported by correlation tests to determine significant predictors of RPA adoption intention. The robustness of the model, which was verified by multiple pre- and post-analysis checks, indicated that institutional support, particularly normative pressure, has the strongest influence on adoption intention, with an adjusted R^2^ value of 0.27 highly significant. This highlights the crucial role that organizational readiness, managerial support, and technology readiness play in enabling RPA adoption. On the other hand, mimetic pressure showed a negative influence, indicating that the industry-wide adoption of RPA technology may raise concerns and anxiety about job displacement. Overall, the findings reinforce the importance of organizational capacity-building in fostering RPA adoption while also revealing the complexity of environmental and technological factors that influence the adoption decisions of professional accountants in a developing-economy context. The findings support SDG 9 by emphasizing capacity building and inclusive digital transformation.

## 1 Introduction

The accounting profession, which has long been grounded in established rules and practices, is now experiencing rapid transformation owing to digitalization and the proliferation of Fourth Industrial Revolution (4IR) technologies (Taib et al., 2023). Among these, Robotic Process Automation (RPA) —a software program that interacts with existing applications to automate repetitive rule-based accounting tasks—artificial intelligence (AI), blockchain, and cloud computing are no longer theoretical concepts for this profession but are now actively reshaping its landscape by automating traditional tasks, invoice processing, bank statement reconciliation, and redefining the skills required for professional processes and outcomes (Judijanto et al., 2025; Kokina and Blanchette, 2019; van der Aalst et al., 2018). RPA tools offer improved efficiency, minimized error, operational cost savings, and provide professionals ample time to focus on high-value analytical and advisory roles instead of the traditional repetitive tasks common to the profession (Doolin et al., 2025; Perdana et al., 2023).

Beyond efficiency, recent research has shown that professional accountants and auditors now integrate RPA with other emerging tools such as machine learning (ML) and generative AI systems to perform intelligent process automation, consequently transforming their roles and skills requirements (Kokina et al., 2025). Furthermore, recent studies find that RPA adoption depends largely on technological, organizational, and environmental (TOE) factors that significantly influence its successful adoption and application within the field of financial accounting (Doolin et al., 2025; Rovaris et al., 2025; Thangapandian et al., 2025). For instance, Rovaris et al. (2025) stressed that technological readiness in the form of perceived usefulness, technological reliability, and system compatibility significantly shapes adoption outcomes. Organizational support in terms of digital culture, training, resource availability, and leadership/management buy-in drives RPA implementation and adoption intention (Thangapandian et al., 2024). Kunene (2023) noted that environmental factors in the form of competitive pressure within the industry, regulatory expectations, supplier support, and client demands create external pressures that shape adoption decisions in accounting firms. Such research demonstrates that behavioral intention toward RPA is not random nor merely a determination by an individual; rather, it is a structured and quantifiable antecedent based on TOE factors.

Despite RPA’s transformative potential and the recent scholarly evidence on how RPA adoption and behavioral intention are shaped by TOE factors, its adoption rate remains uneven across countries and sectors. A report by Fortune Business Insights (2025) recorded that RPA has gained wide recognition in developed nations, with North America leading with the largest market share owing to its large corporate automation investments and government support for digital transformation. They further noted that countries in the Asia-Pacific are recording a rapid growth rate, while the adoption rate in the Middle East and Africa (MEA) is still slow. Similarly, in Cameroon, Fossung and Manfo (2025) reveal that the use of RPA technologies in accounting for rendering auditing services is still under-utilized, with an adoption rate of only 28%, despite firms using other basic accounting tools widely. The rate of adoption in South Africa is, however, yet to be determined, even though the country has been recording gradual growth in digital transformation. In addition, existing studies so far within this context (Kunene, 2023; Mlambo, 2022; Mongwe, 2024; Phage, 2023) have been mainly qualitative in approach and have focused only on financial institutions (banks and insurance companies), while empirical evidence from other sectors is still scarce and fragmented. This existing evidence, though, has failed to report on the level of RPA adoption; such studies have revealed that adoption is largely influenced by skills shortages, governance structures, organizational culture, legacy systems, and job displacement. Another study on the automotive industry within this context produced mixed findings, with some respondents also expressing concern about skills obsolescence and job displacement, but other respondents report efficiency gains (Dlamini, 2024). From the Brazilian credit cooperative system, Rovaris et al. (2025) similarly reported that motivators of RPA include increased operational efficiency, ease of technology use, risk mitigation, cost reduction, and external competitive pressure, while hindrances to its implementation are initial employee resistance and the absence of clear guidelines.

This limited evidence suggests that while RPA offers significant benefits, its adoption is influenced by TOE factors and that its impacts on the roles, skills, job security, and career progression of accounting professionals are far from deterministic. They also highlight a significant void between potential and actual deployment. Such complexity underscores the need for a study from an emerging context, where adoption decisions and the ultimate effect on accounting professionals may be differently shaped by organizational readiness, institutional supports, governance structures, resource constraints, technological capability, regulatory conditions, training, and skills shortages (Da Silva Costa et al., 2022; Moloi and Obeid, 2024; Perdana et al., 2023; Rawashdeh et al., 2022).

Therefore, this study investigates the extent to which RPA technologies are being adopted in South Africa, particularly by accounting professionals, and into factors shaping their behavioral intention to adopt the technology. This is crucial, as the majority of existing studies (such as Kokina et al., 2025; Tiberius and Hirth, 2019; Yang et al., 2024) have been based on developed economies, while the few from South Africa, an emerging country (Dlamini, 2024; Kunene, 2023; Phage, 2023) are largely qualitative and contextual, having their focus on case-based investigations in automotive and financial industries, limiting their generalizability. Additionally, studies that have examined accounting, such as da Silva Costa et al. (2022) and Thangapandian et al. (2024), Thangapandian et al. (2025), employed a systematic literature review approach, while Alassuli (2025) only used descriptive methodology. Empirical evidence with a specific focus on professional accountants from different industries in an emerging context is scarce; likewise, studies based on a quantitative research approach employing the TOE framework are close to non-existent.

Given this, our study investigates the following objectives.

- Determine the extent of RPA adoption among professional accountants in South Africa.
- Assess professional’s perception of RPA technologies for executing accounting tasks.
- Examine which TOE factors influence professionals’ behavioral intention to adopt RPA technologies.

### 1.1 Research questions

RQ1: What is the current level of RPA adoption among accounting professionals in South Africa?
RQ2: How do accounting professionals perceive the TOE implications of RPA technologies in executing accounting tasks?
RQ3: To what extent do TOE factors influence the behavioral intention of accounting professionals in South Africa to adopt RPA?

This study offers both theoretical and practical contributions. Theoretically, by providing empirical evidence on the interplay of the technological–organizational–environmental (TOE) framework, we offer insights into how organizational and technological contexts influence the behavioral intentions of accounting professionals to adopt RPA technologies. The study also examines how environmental attributes stemming from the expectations of normative clients and professional bodies and mimetic pressures within the industry shape the technological readiness and willingness of organizations to adopt emerging technologies, as well as the influence of coercive pressures from government policies. On a practical level, the results will guide firm-level decisions (about training, governance mechanisms, change management, etc.) and policy recommendations (regarding specific institutional supports) that hinder RPA adoption. The study, therefore, considers how the findings may impact innovation-driven policies to advance Sustainable Development Goal (SDG) 9 to build resilient infrastructure, promoting inclusive and sustainable industry development to foster innovation. Through the evidence-based insights from this emerging country context, our study contributes to the growing literature and expands digital transformation discourse in the accounting profession, therefore, providing recommendations for professional accounting practitioners, regulators, and policymakers seeking to promote responsible RPA adoption.

The remainder of this study is organized as follows. Section 2 focuses on the concept explanation of RPA, a review of related literature on RPA, gaps in the literature, and explains the TOE framework. Section 3 presents the methodology, Section 4 provides detailed analysis, results, and discussion, and Section 5 concludes the study.

## 2 Literature review

### 2.1 Robotic process automation in the accounting and auditing profession

RPA involves the use of automated technologies that rely on software to perform tasks that are generally executed by humans. Contrary to popular belief, RPA does not involve the use of physical robots but, rather, bot application programming in the form of software (Kokina and Blanchette, 2019; Moffitt et al., 2018) to perform assigned digital labor at the user interface level following structured input programs/commands (Plattfaut and Borghoff, 2022). Using the software program/command, RPA executes repetitive tasks by interacting with existing applications and user interfaces.

RPA and other automation technologies have, for over a decade, gained popularity in the field of professional accounting and auditing owing to the debate that they enable for structured transaction processing, data extraction, cost reduction, human-error elimination, and time-saving by streamlining repetitive tasks and reconciliations (Lacity et al., 2015; Moloi and Obeid, 2024; Perdana et al., 2023; Sethibe and Naidoo, 2022). Therefore, studies have begun to investigate its adoption, benefits, and contextual dynamics across different organizations and regions.

Recent studies on the value creation of RPA have found that it fosters improved operational efficiency, process standardization, evaluation of the quality and accuracy of information, and cost reduction across key organizational value-chain activities such as internal operations and procurement; it thus has demonstrated that it can add value beyond mere task automation (Durão and dos Reis, 2024; Sethibe and Naidoo, 2022). Similarly, in the accounting domain, RPA’s proven potential regarding its ability to improve accuracy have allowed professionals make informed decisions by offering them time to concentrate on strategic duties and adversarial roles (Doolin et al., 2025; Perdana et al., 2023). It is now considered a core component of digital transformation by most modern organizations and serves as a practical solution to streamlining workflows (e.g., accounting, audit, and payables/receivables) and improving data accuracy (Rovaris et al., 2025; Thangapandian et al., 2025).

### 2.2 Empirical findings on RPA

Evidence has shown an increasing number of RPA adopters among practitioners. However, empirical research differs across industries and contexts. Table 1 below summarizes findings from the extant literature.

**TABLE 1:** Summary of empirical literature.

S/N	Author(s) and year	Country/industry context	Main findings	Methodology/ model/theory used	Gaps/limitations identified
1	van der Aalst et al. (2018)	Germany (developed country); business information systems	Defined RPA, early benefits, and workflow automation potential but no specific findings	Conceptual approach, but no model was employed	Focus was entirely conceptual with no empirical evidence of RPA adoption or implementation. Furthermore, the study was based on pre-AI RPA.
2	Moffitt et al. (2018)	United States (developed country); auditing	RPA enhances audit analytics and efficiency through human error reduction and enhanced business value	Conceptual approach based on RPA for audit automation	Lacks empirical testing, with a focus on early-stage theorizing. In addition, it falls short in capturing the TOE framework as core to RPA adoption.
3	Tiberius and Hirth (2019)	Germany (developed country); auditing sector	Digitization contributes to audit efficiency but increases the need to acquire new skills. Overall, experts noted that digital technologies will not replace auditors but rather provide relief and support.	Delphi study based on a survey of auditing experts. No model was deployed.	Scope was limited to specific technologies, and the experts surveyed were predominantly auditors, excluding other accounting professionals. This could lead to professional bias if caution is not exercised.
4	Kokina and Blanchette (2019)	United States (developed country); accounting	RPA automates repetitive accounting tasks, reduces errors, frees human capacity, and enhances accuracy and reporting quality. However, automation adoption by organizations is purely for the development of scorecard tools to rank tasks, adjust governance structures to capture digital employees, and redefine internal controls. Moreover, the adoption rate is still not uniform due to skills gaps and resistance to automation.	Multiple case study approaches combining survey (qualitative interview) with case studies. Theory of task–technology fit (TTF) and technology-to-performance chain (TPC)	Limited exploratory scope; the sample size was based only on nine interviewed individuals, restricting generalizability. Moreover, it failed in examining the impact of institutional factors and behavioral intention.
5	Syed et al. (2020)	Netherlands and Australia (developed countries); multi-sector	Thematic synthesis covered governance, scalability, and exception handling. Findings revealed that challenges arise from monitoring, process selection, and RPA scaling.	Systematic literature review	No focus on adoption determinants; study lacks profession-specific analysis.
6	Rawashdeh et al. (2022)	Jordan (developing country); internal auditing	RPA creates value but may be hindered by infrastructure and readiness constraints. It also highlighted that RPA adoption in internal audits is driven more by organizational than technological and environmental factors.	Quantitative survey based on TOE framework	The use of an online survey with only internal auditors without considering the opinion of other accounting professionals may limit generalizability. Moreover, it is difficult to ascertain the efficacy of the approach. Hence, there is a need for further investigation.
7	Mlambo (2022)	South Africa (developing country); financial institution	RPA adoption is driven by organizational support, resources, and perceived value.	Qualitative research based on semi-structured interviews. The model used is the technology acceptance model (TAM).	Focused only on the banking sector in South Africa.
8	da Silva Costa et al. (2022)	Portugal (developed country); multi-industry	RPA implementation differs due to firm size, maturity, and area of work. RPA is beneficial for cost savings, improved efficiency, reduced error rate, and reduced working hours. However, RPA barriers often arise due to lack of knowledge and experience, poor documentation, stakeholder resistance, data and security concerns, and data incompatibility.	Systematic literature review	The focus was only on Portugal.
9	Zhang et al. (2023)	United States (developed country); accounting firms	RPA implementation produces productivity gains and enhances governance structure, cycle-time reduction, improved accuracy, and an audit trail.	Descriptive methodology based on case studies. Developed RPA implementation framework (with focus on process selection, design, testing, and governance)	Implementation-focused, not adoption-focused. Therefore, it failed to capture professionals’ perception, institutional supports, and organizational readiness that often shape the level of RPA adoption.
10	Perdana et al. (2023)	Singapore (developed country); accounting and auditing firms	Successful RPA implementation is influenced by organizational readiness and governance frameworks, while RPA benefits include cost reduction, improved accuracy, and efficiency gains. In addition, it highlighted RPA challenges, including staff resistance and integration issues.	Qualitative-based case study research with a focus on the big-four accounting firms.	Implementation focus was on the benefits and challenges of adoption. It falls short in quantifying the behavioral intention behind RPA adoption.
11	Phage (2023)	South Africa (developing country); banking sector	RPA is influenced by technological context: RPA process selection, compatibility, stability, availability, monitoring, and support. Organizational stakeholder collaboration, top management support, and business-led initiative. Environmental context: customer satisfaction, competitive pressure, and regulatory requirements. It also found that banks’ legacy systems strongly influence RPA use for business continuity. TTF factors included task, fit, utilization, and performance.	Qualitative research approach (contextual) study; TOE and task–technology fit (TTF) framework	Focus was based only on banks in South Africa.
12	Kunene (2023)	South Africa (developing country); insurance industry	Management support, organizational readiness, and competitive pressure significantly influence RPA adoption. Positive drivers of RPA acceptance are relative advantage, ease of use, compatibility, management support, competitive pressure, and vendor support. Strategy and government regulations had no significant effect, while organizational skills were viewed as a negative influence. In addition, perceived costs and high complexity act as barriers, whereas effective change management was an enabler of RPA adoption.	Survey analysis using semi-structured interview; TOE framework, diffusion of innovation (DOI) theory, and technology acceptance model (TAM).	The exploratory nature and focus on insurance companies within South Africa and the small sample size of only 20 participants limited its generalizability, suggesting that caution must be exercised in its application to other contexts.
13	Yang et al. (2024)	Australia (developed country), professional services industry	AI adoption is driven by learning, strategic alignment, and client expectations. The study found six factors influencing AI adoption—technology affordances, resource constraints, a firm’s innovation, management approaches and AI readiness, competitive pressure, and regulatory pressure—all of which differ significantly among different firm levels.	A multiple case study approach that combined semi-structured interviews with other documents and TOE	Focused only on the professional services industry, and based on a very small sample of 15 informants. Hence, generalizability is restricted.
14	Mongwe (2024)	South Africa (developing country); banking sector	Organizational culture, legal frameworks, and technology readiness interact closely to shape RPA adoption.	Qualitative mixed methods approach; TOE framework	Geographical context and industry limits make generalization to other contexts difficult.
15	Dlamini (2024)	South Africa (developing country); automotive industry	Mixed findings as participants expressed enthusiasm for RPA, given its potential for efficiency gains and reduced mundane tasks. Others have concerns regarding job displacement and skill obsolescence. Additionally, perception of RPA implementation is linked to organizational culture, leadership support, communication, cost, and usability.	Qualitative research using semi-structured interviews; change theory	Focus was specific to the Gauteng automotive industry, and the limited sample size affects its generalizability. Furthermore, the study was purely qualitative and exploratory in nature, with no statistical testing to back its claims.
16	Moloi and Obeid (2024)	South Africa (developing country); accountants’ perception about AI adoption for financial reporting	Perceptions shaping AI adoption include skills, technical readiness, and trust. However, weak regulatory guidelines weaken automation adoption in African contexts.	Mixed-method approach (quantitative research and descriptive design); TOE framework.	While the study indicates digital adoption attitudes in South Africa and among the accounting profession, its focus was on AI adoption, not RPA.
17	Durão and dos Reis (2024)	Cross-industry organization (country not stated)	RPA enhanced operational efficiency and resource use across the value chain.	Exploratory approach using interviews	The focus was on diverse firms, and only seven interviews were conducted with experts. The exploratory nature of the study and the small sample used necessitate further investigation as the results were not validated by any statistical testing. Together, this limits its generalizability
18	Thangapandian et al. (2024)	The country was not stated, but the focus was on financial accounting firms	Findings provided an updated framework for RPA/AI integration with an emphasis on human parameters	Design science research approach based on literature review, TOE framework, DOI theory, and eight lean waste (ELW) management theory	Focus was on financial accounting firms, with limited empirical testing beyond the proposed frameworks
19	Thangapandian et al. (2025)	India (developing country); accounting and finance	RPA enhances accuracy and reduces labor. Lack of competence and integration are barriers to RPA adoption	Mixed methods based on the design science research approach; TOE framework was employed.	Purely exploratory based on systematic review findings
20	Kokina et al. (2025)	United States (developed country); auditing	AI adoption is hindered by regulatory ambiguity and skill shortages.	Field survey based on interviews with 22 participants—theory not specified	Only captures the state of AI development and current challenges with the adoption of AI in auditing
21	Fossung and Manfo (2025)	Cameroon (developing country); audit firms	RPA adoption at 28%. Organizational readiness, financial constraints, lack of technical expertise, and cybersecurity concerns are major barriers to RPA adoption. They also highlighted the influence of professional bodies and socio-cultural factors on RPA adoption.	Mixed-methods approach involving quantitative and qualitative data gathered via questionnaire, TOE framework, DOI, and institutional theory	Audit-specific and country limitations
22	Judijanto et al. (2025)	Indonesia (developing country); financial services sector	AI significantly enhances accounting performance through rapid reconciliation, consistent data processing, and cost reduction. Found a positive contribution of RPA to accounting task performance and employee satisfaction.	Quantitative survey approach with data involving 150 respondents; TAM and the job demands–resources (JD-R) model	Single industry and country focus. Data used focused mainly on outcomes of RPA adoption but failed to provide deeper insights into employee perceptions and organizational strategies for technology adoption.
23	Rovaris et al. (2025)	Brazil; cooperative credit system	Identified 61 elements as motivators/metrics of adoption across TOE dimensions, stressing technological and organizational factors as the most critical.	Semi-structured interview; TOE framework	Single-case focus on the cooperative credit system in Brazil. Hence, limited generalizability to other broader accounting professions
24	Doolin et al. (2025)	New Zealand (developed country), education sector	Identified five process patterns for RPA implementation: initiation, mobilization, configuration, adaptation, and evaluation. Also found that RPA adoption requires organizational redesign and socio-technical alignment.	Semi-structured interview with multiple actors in a university; process theory	Based purely on RPA implementation in a university with no empirical testing to support the result. Findings cannot be generalized.
25	Alassuli (2025)	Jordan (developing country); banking industry	RPA improves audit efficiency; management support is critical for adoption. RPA systems enhance the internal audit process by reducing operational costs and human error and improving work processes. RPA enables continuous auditing, real-time risk management, and proper reporting. Therefore, it fosters change in the role of internal auditors and, consequently, improves organizational compliance and performance.	Quantitative survey with data gathered via e-questionnaire; no theory/model applied	Limited sample size of 12 commercial banks. Failed to investigate the different experiences and challenges that auditors face in implementing RPA. Focus was also only on the short-term implications of RPA adoption and did not consider longer-term implications such as technology obsolescence, integration of systems, and the changing roles of human auditors.

### 2.3 Gaps in the literature

Overall, existing studies suggest that RPA adoption is growing, emphasizes its benefits, and demonstrates how it can be implemented (Perdana et al., 2023; Zhang et al., 2023). However, they fall short in providing explanations on the critical determining factors of its adoption. For instance, there is limited evidence to explain the behavioral intention behind the adoption of RPA technologies. Likewise, perceptions toward RPA technologies and organizational readiness have been noted as challenges by some studies, but researchers rarely employed behavioral intention as a predictor of RPA adoption when testing empirically. Additionally, existing studies rarely consider institutional support in the form of coercive, normative, and mimetic pressures as influences on technology adoption in professions such as accounting. Significant contextual gaps exist for emerging economies, including the South African accounting landscape, so studies have yet to investigate the dynamics within this context. Our study focused on South Africa to address these gaps by empirically exploring accountants’ perception of RPA and their behavioral intention to adopt it in order to ascertain whether RPA adoption is shaped by technological context, organizational support, and environmental influence. By employing a quantitative exploratory approach, our study offers evidence of behavioral and contextual factors that prior literature has ignored, providing a deep insight into RPA adoption in a professional setting from an emerging economy.

### 2.4 Theoretical underpinning

This study is grounded in the technology–organization–environment (TOE) framework propounded by Tornatzky and Fleischer (1990). TOE recognizes that in a professional organizational setting, technology adoption extends beyond individual perceptions and technology alone; it intersects with technological, organizational, and environmental factors. This framework has been widely applied to explain the adoption of emerging technologies such as AI, blockchain, cloud computing, and other digital technologies. Proponents of this theory argue that adoption behavior is generally shaped by several forces that fall under the umbrella of TOE, and they can either be internal or external forces or both contingencies at the same time. Recent studies have demonstrated that the TOE framework is suitable for modeling RPA adoption since it integrates technological readiness with organizational attributes and environmental influences (Fossung and Manfo, 2025; Thangapandian et al., 2025). This interplay is crucial to understanding the multifaceted influences on the behavioral intention of technology adoption in any professional domain. This is particularly crucial for the accounting profession, which is currently experiencing rapid digital transformation, regulatory shifts, and skills-related pressure arising from the automation of its routine tasks. We believe this framework will offer good ground to theorize and understand how and why professional accountants react to technology adoption.

#### 2.4.1 Technological dimension

The perceived usefulness of RPA technologies can be captured under the lens of the technological features that a new technology possesses as this has a higher likelihood of influencing professional accountants’ adoption intention. The extant literature noted that RPA has the capacity to eliminate errors, save time, improve consistency in accounting task execution, and increase throughput. These attributes suggest that when users of RPA technologies find it useful for effectively completing accounting tasks, they will more likely be convinced of the relative advantage it has to offer, consequently influencing their behavioral intention to adopt it (Durão and dos Reis, 2024; Rawashdeh et al., 2022; Rovaris et al., 2025; Syed et al., 2020). Based on this, we propose the following:

H1 Perceived usefulness of RPA technologies is positively associated with professional accountants’ behavioral intention to adopt RPA.

Additionally, it is also important to state that research has noted that the adoption of RPA technologies is often hindered by high complexity, security challenges, integration issues, or difficulty in operating it (Kunene, 2023; Remlein and Nowak, 2025; Thangapandian et al., 2024). Therefore, if users of RPA technologies find it too difficult to operate, if the RPA technology threatens any form of harm to its users (i.e., exposure to security challenges), or if users perceive the RPA technology to be complex, their level of acceptance or adoption of it will reduce. On the other hand, if users were to find RPA technologies easy to operate, such as being user-friendly, very compatible with current workflows, or causing little or no distortion to current systems/applications, it will be more likely to gain wide acceptance and be adopted. Bearing this in mind, we hypothesize that:

H2 Perceived ease of use of RPA technologies is positively associated with professional accountants’ behavioral intention to adopt RPA.

System compatibility, digital skills and the expertise of users, infrastructural support, and the availability of sufficient IT personnel are critical to demonstrating whether organizations are technologically ready for the uptake of new technologies. This will also affect the perception of professionals working in the organization regarding whether they will support the adoption of the technology. Before venturing into the adoption and implementation of a new technology, organizations must ensure that it offers reliability to users and that there are adequate resources (both human and capital resources) to operate and maintain it to ensure its smooth running. Studies on RPA have shown that technological readiness is a key determinant of its successful adoption and implementation (Rovaris et al., 2025; Thangapandian et al., 2025). This is important because when organizations fail to adequately invest in any new automated technology they are acquiring or fail to implement required structures and allocate necessary resources to ensure the smooth operation of a previous automated technology, users are more likely to not support or even rely on the new system(s) that the organization may propose. Moreover, an individual’s experience with past automated technology may largely influence their perception of other similar automated technologies. For instance, if a previously adopted automated technology fails to fulfill the expectations of the users, they may find it difficult to rely on any other automated system because their previous experience may influence their perception of a similar system in the future. Likewise, where users had previously encountered significant challenges with an automated technology, they suffered a great deal of harm, probably due to their organization not providing adequate support to counter the challenges. This could affect their behavior because they are more likely to perceive the organization as not technologically ready for the uptake of the new automated system. As a result, if a new system is eventually implemented, users may find it difficult to place any reliance on it, given their experience. Therefore, it is presumed that for RPA to function effectively in executing accounting tasks and for users to find it reliable, the system itself must meet or even exceed users’ expectations. Additionally, adequate resources such as IT infrastructure must be available for its maintenance to ensure its smooth running, as well as expert personnel with the requisite skills/expertise to operate. Therefore, we develop the hypothesis that:

H3 Perceived reliability of RPA by professional accountants is positively associated with their behavioral intention to adopt the RPA technologies.

Another important contributory factor to technology adoption is the cost of the technology (Durão and dos Reis, 2024). A capital-intensive technology may not be attractive to firms, particularly those operating in small organizations. Kunene (2023) found that one of the key hindrances to RPA adoption is its higher cost. However, other researchers found that RPA adoption, particularly in accounting, enhances performance through reconciliation and consistent data processing, which consequently reduces operational cost (Alassuli, 2025; Judijanto et al., 2025). This implies that at the core of RPA adoption and implementation is cost, as argued also by Dlamini (2024). Therefore, it is crucial to note that while RPA technologies may be perceived as expensive and while not many organizations can afford them, those who invest in/acquire them most likely do so after careful evaluation of their costs against their benefits. That is, where organizations and individuals see that the benefit that will accrue to them later in the future from investing in a technology or using it far outweighs the cost of the investment now, there is a higher chance that they will adopt the technology and even support it. Moreover, where future benefit outweighs initial costs, it will eventually save an organization or individuals some future costs that would ordinarily not have been saved if such technologies had not been available. This is most likely the reason why studies have found that RPA brings about cost reduction and increases the willingness to adopt (Da Silva Costa et al., 2022; Perdana et al., 2023). Bearing this in mind, we hypothesize the following:

H4 Perceived cost–benefit of RPA by professional accountants is positively associated with their behavioral intention to adopt RPA technologies.

Given the wide debate, it is important to test how the technological dimension may influence the behavioral intention of professional accountants to adopt RPA technologies. Hence, this study explores technological influence using four perspectives: RPA perceived usefulness (i.e., the extent to which RPA technologies enhance tasks efficiency and accuracy), RPA perceived ease of use (i.e., how far professional accountants can use RPA technologies at minimum effort to operate it and not cause distortion in current workflows), RPA perceived reliability (the technical readiness of the organization to possess the requisite skills, availability of supportive infrastructural and the digital competences necessary to operate and automate workflows), and RPA perceived cost–benefit (whether the cost of acquiring RPA is lower than the benefit it will offer). Collectively, we argue that professionals are more likely to adopt RPA technologies and show positive behavioral intention toward its use if they perceive it to be useful, easy to use, reliable, easy to integrate with existing systems and that its future benefit outweighs its costs.

#### 2.4.2 Organizational dimension

The organizational aspect captures the internal firm capabilities, support, and readiness shaping automation adoption. This aspect covers the organizational attributes influencing RPA adoption, especially within accounting domains. At a time when automation is rapidly transforming accounting work processes, organizations must now invest in capital and digital skills. In support of this, some evidence exists for how organizational support, digital training of employees, investment in digital tools and human resources, resource availability for the maintenance of digital tools, and management strategy are critical for the implementation of automated technologies (Fossung and Manfo, 2025; Thangapandian et al., 2024). This is even crucial because technology adoption is hindered by a lack of both financial and human resources (Perdana et al., 2023; Remlein and Nowak, 2025). Given these submissions, we hypothesize the following:

H5 The training resources provided by organizations in support of RPA technologies will create a significant impact on the behavioral intention of professional accountants, thus influencing them to adopt RPA.

Research has also noted that leadership readiness in terms of commitment and top management support is requisite to successful automation transformation. This is so because strategic digital vision, optimal resource allocation, and top-level management buy-in are important for technology deployment—hence, critical enablers of RPA (Moffitt et al., 2018; Rovaris et al., 2025; Zhang et al., 2023). To enable accounting professionals to embrace new technologies and transition from manual work processes to automated systems, organizations must provide the necessary support to ease this transition . Based on this submission, we propose the hypothesis that:

H6 The managerial support provided by organizations for RPA technologies will create a significant impact on the behavioral intention of professional accountants, influencing them positively to adopt RPA.

Furthermore, organizational structure and the standardization of accounting procedures are strong determinants of the readiness of RPA adoption. This is crucial because RPA may often encounter strong resistance or poor implementation, especially in organizations where workflows rely significantly on individual judgment for execution. In fact, several studies have reported this as an issue mostly affecting automation in accounting (Remlein and Nowak, 2025; Rovaris et al., 2025). Therefore, the support that an organization provides to its staff/employees, especially professional accountants and internal auditors who are now required to leverage digital tools to effectively discharge their duties, will be reflected in management’s commitment to digital transformation, training and digital skills development (i.e., whether professionals receive sufficient support to work with automated technologies), and the extent to which workflows permit process automation. To this end, we note that professionals’ perception of and behavior toward automation tools depend on whether their organization supports and encourages them and provides the necessary digital resources to ease their task execution, ensuring smooth workflow and enhanced efficiency/performance. Given this, they will most likely show positive behavior toward automation tools and be willing to adopt them. Given this, we hypothesize the following:

H7 A high level of digital readiness by an organization has a positive influence on the behavioral intention of accounting professionals to adopt RPA technologies.

#### 2.4.3 Environmental dimension

The environmental construct covers external pressures and institutional influences that often shape technology adoption. In the context of a developing country such as South Africa, this will include client expectation and market demand, regulatory support, professional standards, competitive pressure, institutional expectations, industry, and technological trends. These factors can be summarized as three forms of institutional pressure—coercive, mimetic, and normative pressures—which are generally believed to influence the adoption and extent of technology use, especially in the accounting field.

Clients’ expectations for value-added services beyond traditional compliance, or market demand for real-time data and prompt reporting in this era of digitalization, can compel accounting firms to adopt automation technologies to meet client expectations and evolving market demands (Moloi and Obeid, 2024; Timmis, 2025). We, therefore, argue that client expectations and market demand for improved professional services will act as normative pressure to compel accountants to adopt RPA technologies for enhanced accounting services and role delivery (Alassuli, 2025; Rawashdeh et al., 2022; Yang et al., 2024). Hence, we hypothesize the following:

H8 Normative pressure arising from the client’s expectation for digitally enabled accounting services has a significant and positive influence on the behavioral intention of accounting professionals to adopt RPA.

Another crucial institutional force compelling firms to embrace automation is the mimetic pressure formed through broader technological and industry trends the such as use of automation and digital technologies, system integration, and the evolving demands for digital service delivery. This dynamic is reinforced by the increasing interest in integrating RPA with AI, commonly termed as “intelligent process automation,” in the field of accounting and finance (Durão and dos Reis, 2024; Thangapandian et al., 2024; Wang et al., 2025). Firms may adopt RPA to stay competitive in a market, especially if their competitors within the same industry use automation to provide cheaper and prompt services to clients or deliver advisory services. Given this, one can argue that mimetic pressure formed due to the desire to gain a competitive advantage is a core determinant of RPA adoption in accounting. It has also been found by researchers that competitive pressure, such as the need to match industry trends about digital efficiency benchmarks at national, regional, and global levels, can force accountants to adopt automation technologies (Ansari, 2025; Durão and dos Reis, 2024; Rovaris et al., 2025). To this end, we hypothesize the following:

H9 Mimetic pressure existing in the accounting market positively influences accounting professionals’ behavioral intention to adopt RPA.

Furthermore, the activities of accounting professionals regarding auditing and financial reporting are often subject to stringent regulatory requirements, compliance standards, and high expectations from professional bodies, especially when operating in a well-regulated setting. Professionals may, therefore, find RPA a desirable alternative when the regulatory environment in which they operate requires them to adopt such technologies for improved accuracy and transparency in reporting. This form of regulatory requirement highlights how professionals can be coerced into adopting automation owing to increasing digital expectations from regulatory authorities. Numerous studies identify coercive or regulatory pressure as a motivator of RPA (Hsiung and Wang, 2022; Rovaris et al., 2025; Thangapandian et al., 2024). In an environment such as South Africa that is currently undergoing significant digital transformation, this form of institutional pressure may be instrumental in fostering the adoption of RPA technology. On this basis, we propose the following hypothesis:

H10 Coercive pressure due to stronger regulatory compliance has a positive influence on the behavioral intention of accountants to adopt RPA.

**FIGURE 1:**
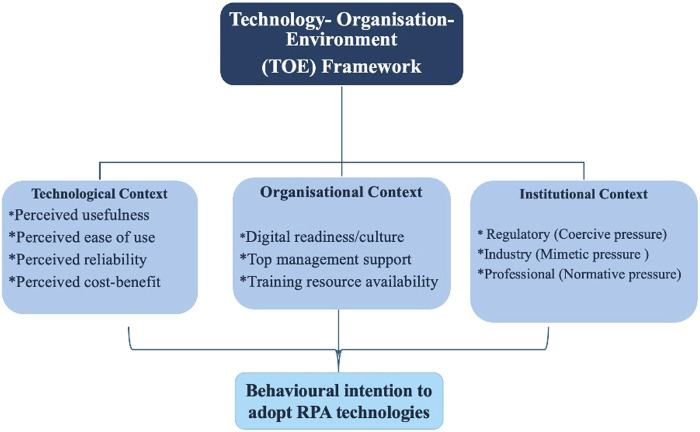
Conceptual framework of RPA adoption based on TOE perspective.

This integrated approach is suitable for our study as it allows for a multidimensional understanding of RPA adoption by South African accounting professionals and firms, extending beyond individual perceptions and mere technology, to align with the organizational and institutional environment in which the accounting profession operates. This, combined with behavioral intention as an outcome variable, can indicate professionals’ readiness to adopt RPA. Moreover, recent studies such as Perdana et al. (2023) and Zhang et al. (2023) recommend the TOE framework as a strong model that any study on digital transformation ought to consider, including studies from the accounting field, because it captures both internal and external factors that shape innovation outcomes. Therefore, by employing this framework for our study situated in the accounting field in an emerging context such as South Africa, the study offers a comprehensive insight into the factors influencing accountants’ behavioral intention to adopt RPA technologies and use them for executing their tasks. Drawing on this, we conceptualize RPA adoption in Figure 1.

## 3 Methodology

### 3.1 Research design, population, sample technique, and instrument

This exploratory study employed a quantitative, cross-sectional survey design approach, allowing for statistical testing of the association between the constructs examined and accounting professionals’ perception of RPA adoption at a particular time. The study targeted accounting professionals working in different areas of specialization within South Africa. However, because there is no central database of accountants within South Africa, a purposive sampling technique was employed to reach the targeted groups via social media platforms (LinkedIn and WhatsApp) with verified user profiles displaying details of the individuals’ professional status and role and through professional associations such as the South African Institute of Chartered Accountants (SAICA), South African Institute of Professional Accountants (SAIPA), and the Institute of Internal Auditors South Africa (IIA SA). This ensured that only qualified professionals in South Africa were invited. Through these media we distributed the questionnaire developed on Google Forms to the targeted groups with a detailed explanation provided on the purpose of the research, eligibility criteria for the individuals to determine whether they are eligible to respond, a confidentiality statement, consent, and estimated completion time. Furthermore, the study had some questions to screen respondents; through the screening responses to the survey questions, we were able to confirm the employment status of the respondents, their designated roles, location in South Africa, basic awareness of automation technologies, and an assessment of their RPA usage with response rated (use: never, rarely, moderately, frequently, or always). This assessment further allowed us to separate the active users of RPA technologies from non-active users. This approach was deemed appropriate given the exploratory nature of the study and the need to obtain information-rich cases from professionals who are knowledgeable about RPA adoption, implementation, and challenges often faced by organizations seeking automation.

#### 3.1.1 Respondents’ composition and sample size justification

The study population was indeterminable, so invitations were sent across the media noted in the preceding section, and participation was voluntary. The study recorded an initial response of 127, comprising both active and non-active users of RPA technologies as recorded in the self-assessment screening question. Therefore, to capture only responses of actual RPA users for further analysis, we screened out non-active users (27 in total) to arrive at the final sample of 100 responses from active users. The respondents in the group worked in various organizations with roles across finance, accounting and audit, taxation, public sector, insurance, and technology service providers supporting RPA deployments. The final sample of 100 responses was deemed sufficient for this study as this is within the recommended minimum acceptable threshold of Hair et al. (2010).

#### 3.1.2 Research instrument

The structured questionnaire (see appendix) was composed of items on a five-point Likert scale: strongly agree = 5, agree = 4, neutral = 3, disagree = 2, and strongly disagree = 1. The construct comprised technological, organizational, and environmental contexts, with behavioral intention as outcome variable, and adoption level to ascertain the extent of adoption in South Africa. Table 2 summarizes the final items used in the study analysis.

**TABLE 2:** Survey construct employed for data preparation and analysis.

Pre-assessment item	
RPA usage	How would you describe your level of robotics usage on a scale from 1 (never used) to 5 (always used)?
RPA knowledge	How would you rate your knowledge of robotics on a scale from 1 (very poor) to 5 (very good)?
Organization/role	Voluntary open-ended responses requiring the respondents to state their organization or their designated roles.
RPA adoption level (yes/no responses)	
	Please kindly signify your organization’s adoption category.
Category A	My organization currently uses robotics for the processing of its activities and execution of accounting tasks.
Category B	My organization plans to adopt robotics during the year.
Category C	My organization is not using and has no plans to use robots in the near future.
Behavioral intention (outcome variable)	
BI1	I intend to use robotics to perform daily accounting tasks in the future.
BI3	I intend to learn how to use robotics soon.
Technological context	
Variable code Technology dimension A: perceived usefulness	
TU 1	I believe that a robot would enable professional accountants to accomplish tasks more quickly.
TU 2	Using a robot to perform tasks would save me a lot of time at work.
TU 3	I believe that using robots would improve my performance by reducing errors.
TU 4	Robots will increase the value that I provide in my work.
TU 5	Having robots would allow me to use my time to accomplish other tasks.
TU 6	Using a robot will make my work easier and more efficient.
Technology dimension B: perceived ease of use	
TEOU	Applying robotics to my daily accounting tasks would be easy for me.
Technology dimension C: perceived reliability	
TR	I believe that robots will provide value when performing tasks.
TR	I would trust the results provided by the robot when performing tasks.
TR	I am interested in learning about robotics and how to apply it in accounting and auditing jobs.
TR	I believe that robots will provide reliable results for tasks that are repetitive tasks better than humans.
Technology dimension D: perceived cost–benefit	
TCB	I believe that using robotics to perform daily tasks will reduce costs.
Organizational context	
DR	My organization supports the use of robots for professional accounting services (digital readiness).
MS	My manager encourages and supports the use of robots in performing accounting work (managerial support).
TRes	My organization has established internally good structures and training resources that can support my learning and use of robotics.
Environmental/institutional context	
NP	The expectations of my clients and professional accounting bodies are that professional accountants should use robotics in performing routine work or audits (normative pressure).
CP	The South African national government has policies and initiatives that encourage/support the adoption of RPA technologies by accounting professionals (coercive pressure).
MP	Other firms within the industry have adopted RPA technology, and there are industry-wide resources to support its use for accounting processes (mimetic pressure).

### 3.2 Instrument reliability (Cronbach alpha)

The study adapted a validated instrument (see Appendix) used in previous technology adoption studies (Attuquayefio and Addo, 2014; Kim et al., 2024). We assessed the internal consistency of the instrument using Cronbach’s α (Table 3) where necessary because some constructs had multiple items, while others had only a single item. The combined alpha was 0.8. Under the technological dimension, we had constructs measuring technology perceived ease of use (TEOU), technology perceived usefulness (TU), technology perceived reliability (TR), and technology perceived costs–benefits. For our multi-item constructs (TU and TR), we followed standard psychometric guidance (Nunnally and Bernstein, 1994; Tavakol and Dennick, 2011). Table 3 summarizes the results of the reliability, validity testing, and exploratory factor analysis. TU with α = 0.778 demonstrated good internal consistency and was retained with its composite mean score (Hair et al., 2010). TR showed an alpha value of 0.63. While this is slightly below the conventional 0.70 threshold, research noted that an α value of ≈0.60 is acceptable for exploratory scale development and early-stage research (Nunnally and Bernstein, 1994; Taber, 2018). Hence, based on the acceptable values of the item-total correlations and a single-factor structure that the four item scales produced, the mean composite score was retained. Behavioral intention, BI1 and BI3, produced α = 0.503 when combined. Eisinga et al. (2013) noted that the reliability indices of two-item scales are sometimes lower due to scale length but are conceptually coherent. All other constructs were regressed separately and interpreted individually, allowing for more precise interpretation. All analyses covering both descriptive and inferential statistics were conducted in SPSS version 29.

**TABLE 3:** Reliability, validity, and factor analysis output table.

Construct	Items (N)	Cronbach’s α	KMO	Bartlett’s χ^2^	Single factor variance	Composite mean	SD
TU	6	0.778	0.773	χ^2^ (15) = 169.62***	35.7%	3.40	0.86
TR	4	0.628	0.679	χ^2^ (6) = 46.48***	31.0%	3.38	0.88
Beha_Int13	2	0.503	-	-	-	3.17	1.03
Combined	27	0.80	0.81	-	-	-	-

Significant level ***. (1%), ** (5%), * (10%).

### 3.3 Instrument validity

A KMO value of 0.81 (Table 3) indicated sampling adequacy, and Bartlett’s test was significant (*p* < 0.001), confirming factorability. Exploratory factor analysis using Promax rotation revealed a clear four-factor structure consistent with the TOE theoretical model. TU (6 items) with KMO = 0.773, Bartlett’s χ^2^ (15) = 169.62, and *p* < 0.001 showed a single factor explaining 35.7% of variance (α = 0.778). TR (four items) also with KMO = 0.679, Bartlett’s χ^2^ (6) = 46.48, and *p* < 0.001 had one factor explaining 31% variance (α = 0.628), both suggesting their suitability and acceptability for exploratory research (Nunnally and Bernstein, 1994; Taber, 2018). All reflective items loaded strongly (0.53–0.82) on their respective dimensions with no problematic cross-loadings. Behavioral intention items clustered together as expected. Institutional support indicators were excluded because they represent conceptually distinct pressures (normative, coercive, and mimetic) and are treated as separate theoretical variables. Overall, the results confirm the construct validity of the measurement instrument.

The regression model employed is

$$\begin{array}{ll} BI1\_3 & =\beta_{0}+\beta_{1}\left(TEOU\right)+\beta_{2}\left(TU\right)+\beta_{3}\left(TR\right)+\beta_{4}\left(TCB\right)+\beta_{5}\left(DR\right)\\ & \;+\beta_{6}\left(MS\right)+\beta_{7}\left(TRes\right)+\beta_{8}\left(NP\right)+\beta_{9}\left(CP\right)+\beta_{10}\left(MP\right)+\epsilon . \end{array}$$

### 3.4 Ethical consideration

All respondents provided their consent to participate in the study and were assured of their anonymity. The study also received ethical approval with code SAREC20241024/05.

## 4 Result presentation, interpretation, and discussion

### 4.1 Pre-test diagnosis and normality assessment

Before proceeding with the analysis, we checked for missing items; none were missing. We also checked to detect outliers, but only two cases were flagged as mild outliers; since these were valid responses, we retained them in the analysis. Normality was assessed using the Shapiro–Wilk test and Q–Q plots. Both Kolmogorov–Smirnov and Shapiro–Wilk tests were significant (*p* < 0.05), indicating an over-sensitivity to minor deviations, especially given that the study sample size exceeded 50 observations (Ghasemi and Zahediasl, 2012; Shatz, 2024). Hence, it has been noted that this is not an indication of non-normality when taken alone (Arnastauskaitė et al., 2021; Avram and Măruşteri, 2022). Using recommendations from recent literature offering methodological guidance, we plotted the Q–Q plots for visual inspection of the data distributions. The Q–Q plots (Supplementary Appendix A1), however, showed that the distribution of all items was closely aligned to the diagonal line with no substantial deviations, indicating that the distributions were approximately normal. Hence, they were suitable for regression-based analyses that are robust to mild departures from normality (Ghasemi and Zahediasl, 2012; Shatz, 2024). On this basis, we proceeded with the parametric testing.

### 4.2 Result analysis and interpretation

#### 4.2.1 What is the current level of RPA adoption among accounting professionals in South Africa?

Table 4 below provides a summary of the RPA adoption level among accounting professionals in South Africa.

**TABLE 4:** RPA adoption indicators.

RPA adoption level	No of adoptions	Percent
Category A: Organizations currently using RPA	37	37%
Category B: Plans to adopt RPA	30	30%
Category C: No intention to adopt RPA	33	33%
Total	100	

The results indicate a varying level of RPA adoption among the accounting professionals in South Africa. Table 4 shows that 37% of the respondents noted that their organizations are currently using RPA, while 30% planned to adopt RPA within the year and 33% had no plans of adoption. Overall, approximately 63% of respondents could be categorized as prospective adopters and non-adopters of RPA. This suggests a relatively low momentum of RPA adoption for accounting and auditing processes by South African accountants, though moving gradually toward aligning with global digital transformation trends. This answers the first research question and confirms the recent report that RPA adoption level is still low in the African region (Fortune Business Insights, 2025).

#### 4.2.2 What are accounting professionals’ perceptions about RPA technologies for executing accounting tasks?

The results from Table 5 had values that demonstrated some organizational readiness and generally mild-to-moderately positive perceptions of RPA’s technological features; the weakest from all constructs is MS (managerial support), which had a mean of 2.91(SD = 1.26) under the organizational context. This implies the respondent’s perception that management providing necessary support for automation uptake is very low. Most likely, management support is not yet visible or not available in most organizations. For the technological context, TU had a mean of 3.40 (SD = 0.86), indicating that respondents had a mildly positive perception of RPA usefulness for accounting tasks. TR had a mean of 3.38 (SD = 0.88), indicating that respondents generally agreed that RPA would be reliable for repetitive tasks. TEOU had a mean of 3.22 (SD = 1.17), reflecting modest agreement that RPA is easy to use. PCU had a mean = 3.34 (SD = 1.27), revealing that respondents generally agreed that RPA would offer some benefits that would save costs in the future when it is adopted. For organizational context, DR averaged 3.24 (SD = 1.14), indicating modest agreement by respondents that organizations are digitally prepared for automation. TRs had a mean = 3.01 (SD = 1.1,4), also suggesting modest agreement that organizations provide training resources to expose professionals to RPA. For constructs under the environmental/institutional context, NP had a mean value of 3.03, CP had a mean value of 2.94, and MP’s mean = 2.98, with their SDs ≈1.16–1.24 collectively reflecting a neutral to slightly positive perception of institutional pressures.

**TABLE 5:** Descriptive statistics.

Variable	N	Minimum	Maximum	Mean	Standard deviation	No. of items
TEOU	100	1	5	3.22	1.17	1
TU	100	1.00	5.00	3.40	0.86	6
TR	100	1.00	5.00	3.38	0.88	4
TCB	100	1	5	3.34	1.27	1
DR	100	1	5	3.24	1.14	1
MS	100	1	5	2.91	1.26	1
TRes	100	1	5	3.01	1.14	1
NP	100	1	5	3.03	1.23	1
CP	100	1	5	2.94	1.16	1
MP	100	1.0	5.0	2.98	1.24	1
BI1_3	100	1.00	5.00	3.17	1.03	2
Valid N	100					

N implies total number of valid responses.

The bivariate associations revealed in Table 6 show that BI1_3 (behavioral intention) had a positive and significant correlation with all constructs except Tres, CP, and MP. The largest and most meaningful is found with NP (normative pressure). The favorable mean values and significant positive correlation that TU and TR had with BI suggest that professional accountants perceive that RPA technologies can be beneficial, useful, eliminate errors, and are reliable for repetitive tasks, consistent with previous research (Durão and dos Reis, 2024; Rawashdeh et al., 2022; Rovaris et al., 2025; Thangapandian et al., 2025).

**TABLE 6:** Correlation matrix.

	BI1_3	TEOU	TCB	TU	TR	DR	MS	TRes	NP	CP	MP
BI1_3	1										
TEOU	0.29***	1									
TCB	0.36***	0.1	1								
TU	0.31***	0.33***	0.43***	1							
TR	0.36***	0.35***	0.41***	0.54***	1						
DR	0.30***	0.07	0.34***	0.27***	0.29***	1					
MS	0.27***	0.20**	0.11	0.21**	0.22**	0.19*	1				
TRes	0.16	0.23**	0.08	0.28***	0.30***	0.30***	0.15	1			
NP	0.39***	0.31***	0.27***	0.32***	0.33***	0.08	0.11	0.23**	1		
CP	0.15	0.14	0.19*	0.16	0.06	0.13	0.34***	−0.04	0.19*	1	
MP	0.05	0.21**	0.11	0.12	0.10	0.15	0.35***	0.34***	0.29***	0.25**	1

Significant level ***. (1%), ** (5%), * (10%).

Overall, all three TOE contexts reveal that professionals have a relatively positive perception of RPA technology, although their perception was uneven across all contexts. For instance, perception about NP (normative pressure from clients) showed the most meaningful factor likely to influence BI, followed by TR, TCB, and TU. This implies that the reliability of a technology, presumed cost–benefit (future cost saving), and perceived usefulness are all very crucial in determining intention to adopt and use RPA for executing accounting tasks. This aligns with the TOE framework that a single construct does not by itself influence behavioral intention to adopt new technologies; rather, a triad of factors is a key determinant (Dlamini, 2024; Tornatzky and Fleischer, 1990). It is also consistent with extant research (Durão and dos Reis, 2024; Fossung and Manfo, 2025; Perdana et al., 2023; Rovaris et al., 2025). Meanwhile, the individual factors might not drive intention to use RPA because the level of influence they exert alone as a single construct might not be sufficient compared to when they all work together. A correlation heatmap is included in the appendix.

#### 4.2.3 To what extent do TOE factors influence the behavioral intention of accounting professionals in South Africa to adopt RPA?

**FIGURE 2:**
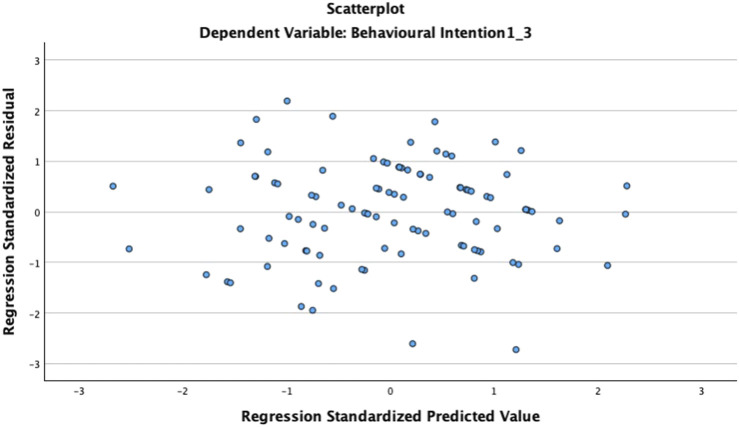
Scatterplot of residuals with BI as dependent variable.

The predictive contribution of our TOE variables on behavioral intention was tested using multiple regression analysis (see output in Table 7). The final model explained R^2^ = 34.3% of the total variance in the BI composite, with adjusted R^2^ = 27%. Overall, the model was significant, F (10, 89) = 4.650, *p* < 0.0. Cohen’s f^2^ for assessing overall effect size for the model based on R^2^ = 0.343 is 0.522, implying a large effect size for the predictor variables collectively. However, after accounting for the sample size and number of predictors, the adjusted R^2^ value (≈0.27) shows that roughly 27% of the variance in behavioral intention is explained by the predictors. Multicollinearity was tested with VIF (values <2) and tolerance (values > 20), both indicating no multicollinearity issues. Heteroskedasticity was also assessed using the Breusch–Pagan (BP) test, *p* = 0.24, and scatterplots (see Figure 2) with residuals approximately revealing random dispersion. Both results showed no significant heteroskedasticity, indicating the error variance across the observations was constant. Furthermore, we assessed the linearity assumption. The Durbin–Watson value of 2.24 was within the acceptable threshold, indicating no autocorrelation or linearity assumption violations. Overall, all diagnostic tests confirmed that the study data met the essential assumptions for multiple regression analysis, strengthening the validity and reliability of the model.

**TABLE 7:** Multiple regression output (OLS).

Predictor	Coefficient (B)	Standard error	t	β	Tolerance	VIF
Constant	0.773	0.481	1.607			
TEOU	0.137	0.086	1.585	0.155	0.776	1.288
TU	−0.023	0.133	−0.174	−0.019	0.600	1.666
TR	0.069	0.133	0.519	0.058	0.581	1.722
TCB	0.155*	0.085	**1.821**	0.189	0.682	1.466
DR	0.158*	0.089	**1.786**	0.174	0.775	1.290
MS	0.182**	0.081	**2.250**	0.222	0.759	1.318
TRes	0.005	0.092	0.058	0.006	0.712	1.404
NP	0.250***	0.084	**2.986**	0.297	0.746	1.341
CP	−0.015	0.086	−0.173	−0.017	0.792	1.263
MP	−0.163*	0.085	**−1.922**	−0.196	0.711	1.406
Model fit						
R	0.586					
R-squared	0.343					
Adjusted R^2^	0.269					
F (10, 89)	4.650***					
Durbin–Watson	2.244	No autocorrelation				
Breusch–Pagan	0.24	No heteroskedasticity				
DV	Behavioral intention (BI1_3)					
Cohen’s f^2^	0.522					

Significance level ***. (1%), ** (5%), * (10%).

Model fit bolded refers to the post estimation tests conducted to enhance the robostness of the results.

From the regression output (Table 7), variables with the most significant influence were normative pressure (NP), a construct under institutional context (β = 0.297; *p* = 0.004) having the strongest predictor, followed by managerial support (MS) (β = 0.222; *p* = 0.027), also exhibiting substantive influence under organizational context. Digital readiness (DR) and RPA perceived cost-benefit (TCB) have β = 0.174 and p = 0.078 and β = 0.189, p = 0.072, respectively, both implying marginal influence. Mimetic pressure (MP) (β = −0.196; p = 0.058) showed a mild negative influence. However, despite RPA’s usefulness (TU) and reliability (TR) having a significant positive bivariate correlation with BI (Table 6), they did not exhibit independent predictive power in the regression model.

The visual display in Figure 2 is the scatterplot from the regression output. A close examination of the standardized residuals showed no evidence of non-linearity or heteroscedasticity. The residuals were randomly dispersed around zero, with a relatively constant spread across the predicted values. This suggests that all the assumptions of linearity and homoscedasticity were met as previously mentioned. The non-significant value of the BP test further supports this assumption, indicating that the behavioral intention is not distorted by any violations. Overall, this shows the reliability of the model interpreted. Hence, we are confident that all the key assumptions of linear regression are satisfied.

### 4.3 Discussion

The positive effect of NP implies that professional accountants are highly likely to use RPA technology if they perceive high expectations from clients, professional associations, or based on higher market demand for value-enhanced services. Normative pressure tends to grant legitimacy and normative approval by reducing perceived social risk and increasing behavioral adoption intention. This finding is consistent with the environmental context of TOE that normative pressures are central to the regulatory environment or institutional level shaping organizational and professionals’ actions. It also adds to prior TOE studies that document institutional influence in the form of normative pressure are enablers for RPA technology adoption in professional domains such as accounting (Alassuli, 2025; Rawashdeh et al., 2022; Yang et al., 2024). Moreover, clients’ expectations for value-added services beyond traditional compliance and market demand for real-time data significantly shape the adoption of accounting and auditing technologies in this digitalization era (Moloi and Obeid, 2024; Timmis, 2025). Hence, we accept H8: “Normative pressure arising from clients’ expectation for digitally enabled accounting services has a significant and positive influence on the behavioral intention of accounting professionals to adopt RPA.”

This finding also links to SDG 9 by shaping professional values toward innovation-driven practices. Normative pressure exerted on professional organizations can hasten the adoption of digital technologies such as RPA that enhance data-driven services, industrial efficiency, and innovation diffusion, thereby advancing SDG9 targets for resilient infrastructure and innovation.

The positive significant effect of managerial support (MS) implies that support provided by top management of an organization in terms of management buy-in, leadership readiness in terms of commitment and encouragement, or even sponsor-led advocacy will significantly influence behavioral intention. Additionally, management support through strategic priority-setting and investment in human and capital resources can signal to professional accountants that the organization supports automation. This will influence their perception and reduce any barrier to automation, consistent with Moffitt et al. (2018), Rovaris et al. (2025), and Zhang et al. (2023). The findings align with the organizational context of TOE that management support is an essential element of organizational readiness. On this basis, we accept H6 that managerial support provided by organizations for RPA technologies will create a significant impact on the behavioral intention of professional accountants, influencing them to adopt RPA. Furthermore, if NP, in terms of professional standards and guidance, is now combined with investment decisions under MS, they can both act as enablers needed to scale RPA adoption, foster industrial processes, innovation capability, and productivity, linking with the core aims of SDG 9.

The positive, significant but marginal influence exhibited by digital readiness (DR), a component of organizational context with BI, also reflects that when firms demonstrate that they are prepared for digital uptake, it will increase the likelihood that professional accountants will use RPA tools. This aligns with the organizational context of TOE, which noted that organizational digital readiness is a key determinant of automation adoption (Tornatzky and Fleischer, 1990). It is also consistent with prior evidence that leadership readiness in terms of commitment and top management support is requisite to successful automation transformation (Moffitt et al., 2018; Rovaris et al., 2025; Zhang et al., 2023). Hence, we accept H7 that a high level of digital readiness by an organization has a positive influence on the behavioral intention of accounting professionals to adopt RPA technologies. This result has implications for organizations. It encourages them to pair technical upgrades with management strategy and investment in core IT infrastructures and technology integration capabilities before implementing RPA on a large scale throughout the organization. This also links directly to SDG9 that digital readiness can foster technology diffusion and innovation uptake.

Furthermore, the marginally positive significant effect also revealed by RPA TCB, reflective of professionals’ perception and intention to use RPA adoption, is that it reduces cost and enhances efficiency. This finding aligns with the TOE technological context that cost–benefit/relative advantage is a core component of the technological context. It is also consistent with prior submissions that cost is essential for technology adoption (Dlamini, 2024; Durão and dos Reis, 2024), particularly in accounting and professional services, because it enhances performance through reconciliation and consistent data processing (Alassuli, 2025; Judijanto et al., 2025). With this finding, we accept H4 that the perceived cost–benefit of RPA by professional accountants is positively associated with their behavioral intention to adopt RPA technologies. The findings also align with SDG 9’s goals for a resilient, competitive industry, noting that the cost–benefit accruing from automated technologies can foster industry efficiency and boost productivity.

The negative effect from mimetic pressure (MP) suggests that professionals’ perception of automation uptake by their peers/competitors in the industry might drive fear and anxiety of job loss or insecurity, consistent with prior submissions that automation and increased digitalization can cause job losses in accounting domain (Dlamini, 2024; Mlambo, 2022; Thangapandian et al., 2025) and consequently reduce professionals’ willingness to adopt RPA technologies. Based on this finding, we reject H9 that mimetic pressure existing in the accounting market positively influences accounting professionals’ behavioral intention to adopt RPA. The implication is that industry imitation alone may not always be sufficient to encourage professional accountants to use RPA tools. As a matter of fact, it may even discourage them, especially when they fear job displacement due to automation.

## 5 Conclusion, research implications, and limitations

### 5.1 Conclusion

This study examined the perception of professional accountants in South Africa and their behavioral intention toward adopting RPA technologies, examining this relationship through TOE factors. The findings indicate that while perceptions about the value that RPA offers are generally positive, organizational and environmental (institutional) factors play a crucial role as they exert a stronger influence on actual adoption intention. Of all the TOE factors, normative pressure showed the strongest positive influence on behavioral intention to adopt RPA, while mimetic pressure revealed a significantly negative influence. This is indicative that beyond mere imitation within industry, professionals might have concerns with job displacement due to automation, and thus are not encouraged to support automation adoption. Overall, the model explained 27% of the variation in behavioral intention, indicating that beyond TOE factors, adoption decisions are also influenced by psychological factors.

The study, therefore, contributes novel evidence by applying the TOE framework to examine RPA adoption within an emerging economy professional accounting context. It challenges conventional assumptions in technology-adoption models by demonstrating that technological factors can have detrimental effects when perceived usefulness and perceived threat coexist. The findings that adoption may be discouraged by mimetic pressure further elucidate the limits of environmental influence in a professional, autonomy-driven industry such as accounting.

SDG 9 also strengthens this study’s theoretical claim that digital transformation is part of the broader developmental agenda rather than a mere change in technology. The findings, therefore, position RPA adoption as both an imperative of society and an organizational decision for fostering sustainable industry innovation.

### 5.2 Implication of findings and recommendation

Basically, the findings of this study strongly support prioritizing institutional support (normative pressure) along with management support, digital training, and technical resources because they are the most effective determinants that shape adoption. Mimetic pressure, negative influence, and the non-significance of technological support factors reinforce the need for structural change in management strategies to allay professionals’ fears and anxiety about job displacement. It is also important for professional bodies and organizations to encourage industry-specific RPA readiness guidelines rather than depending on competitive imitation since mimetic pressures fail to drive adoption intention. These recommendations align with SDG 9’s campaign for inclusive digital transformation and capacity building.

### 5.3 Limitations and suggestions for future research

This study is not without some limitations. A common method bias that is often introduced when a study relies on self-reported perception data limits causal inference. Moreover, the small sample size also limits generalizability. Additionally, the low adjusted R^2^ of 27% suggests that some key variables, such as organizational culture, technological anxiety, and perception regarding threats to the job, were not captured, and they might have a stronger influence on actual intention to adopt technologies. Future research is encouraged to use a longitudinal approach by integrating other organizational constructs not captured in this study and psychological factors.

## Data Availability

The raw data supporting the conclusions of this article will be made available by the authors, upon reasonable request.
