# Supplementary material for: Understanding accounting professionals’ intention to adopt robotic process automation: a TOE-based empirical assessment from an emerging country
Source: Front Robot AI. 2026 Jan 29;12:1747539. doi: 10.3389/frobt.2025.1747539 (PMC12895344; doi:10.3389/frobt.2025.1747539)
Supplement: Supplementary file 1 [file Supplementaryfile1.docx]

**Appendix**

**Appendix A1:** Pre-test Q-Q plots


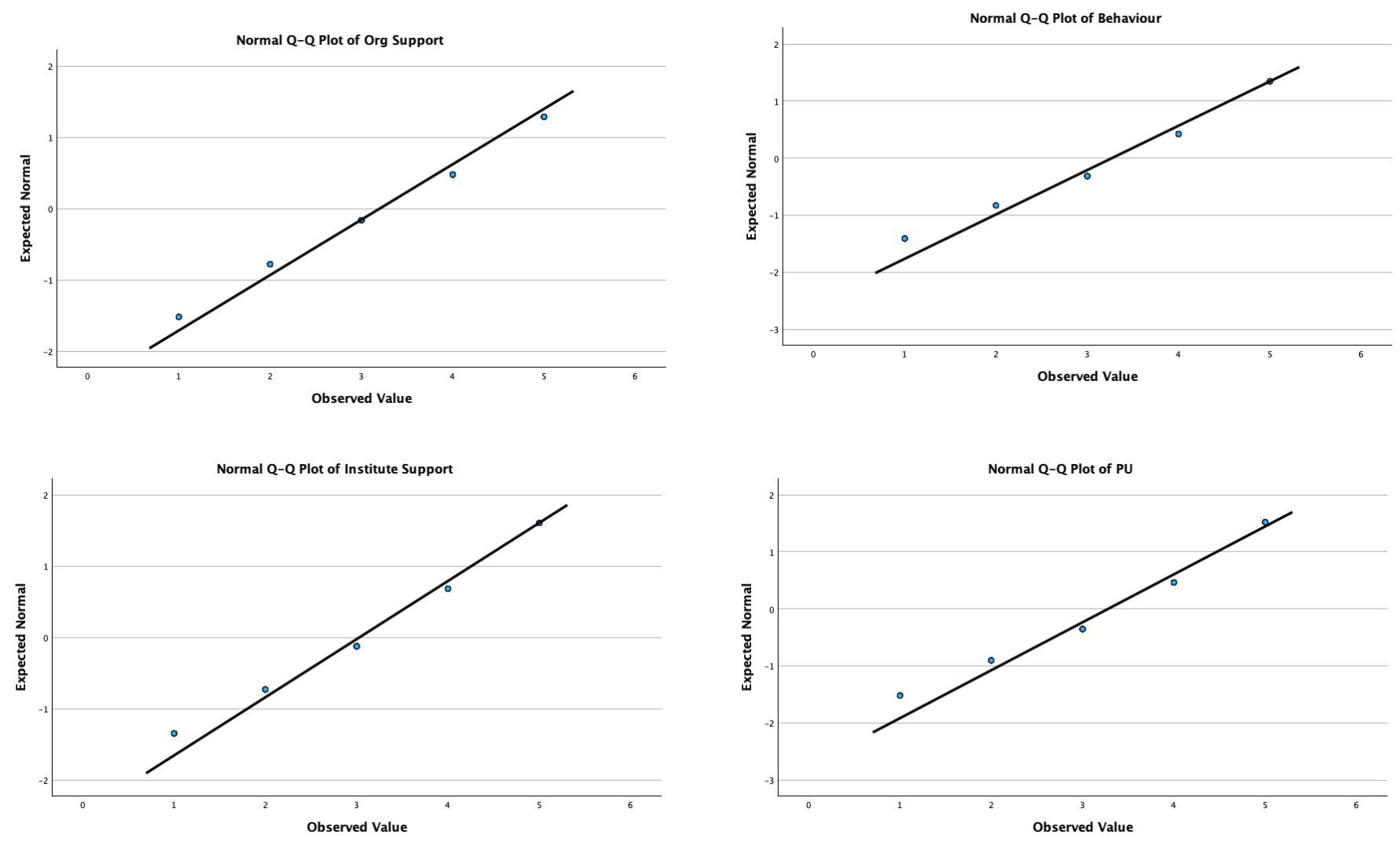


**Appendix Table A2 for Factor Communalities on RPA Usefulness**

| **Item** | **Initial** | **Extraction** |
| --- | --- | --- |
| Tech5 | .336 | .373 |
| Tech6 | .309 | .276 |
| Tech7 | .330 | .238 |
| Tech8 | .437 | .580 |
| Tech9 | .340 | .375 |
| Tech10 | .334 | .379 |
| Tech11 | .271 | .275 |

**Appendix Table A3: Correlation Matrix Heatmap**

|  | BI1_3 | TEOU | TCB | TU | TR | DR | MS | TRes | NP | CP | MP |
| --- | --- | --- | --- | --- | --- | --- | --- | --- | --- | --- | --- |
| BI1_3 |  | -- |  |  |  |  |  |  |  |  |  |
| TEOU | 0.29*** | 1 |  |  |  |  |  |  |  |  |  |
| TCB | 0.36*** | 0.1 | 1 |  |  |  |  |  |  |  |  |
| TU | 0.31*** | 0.33*** | 0.43*** | 1 |  |  |  |  |  |  |  |
| TR | 0.36*** | 0.35*** | 0.41*** | 0.54*** | 1 |  |  |  |  |  |  |
| DR | 0.30*** | 0.07 | 0.34*** | 0.27*** | 0.29*** | 1 |  |  |  |  |  |
| MS | 0.27*** | 0.20** | 0.11 | 0.21** | 0.22** | 0.19* | 1 |  |  |  |  |
| TRes | 0.16 | 0.23** | 0.08 | 0.28*** | 0.30*** | 0.30*** | 0.15 | 1 |  |  |  |
| NP | 0.39*** | 0.31*** | 0.27*** | 0.32*** | 0.33*** | 0.08 | 0.11 | 0.23** | 1 |  |  |
| CP | 0.15 | 0.14 | 0.19* | 0.16 | 0.06 | 0.13 | 0.34*** | -0.04 | 0.19* | 1 |  |
| MP | 0.05 | 0.21** | 0.11 | 0.12 | 0.10 | 0.15 | 0.35*** | 0.34*** | 0.29*** | 0.25** | 1 |
| N | 100 |  |  |  |  |  |  |  |  |  |  |

Significant level ***. (1%), ** (5%), * (10%)

**Questionnaire Items**

|  | **Demographic Data** |
| --- | --- |
| Age | Below 20; 21-30; 31-40; 41-50; 51-60; above 60 |
| Gender | Male; Female; Prefer not to disclose |
| Qualification | Diploma; matric; Bachelor’s degree; Honours; Masters; Doctorate; Others |
| RPA Knowledge | How would you describe your knowledge of robotics on a scale of 5 for Very Good to 1 for Very poor |
|  | **Adoption Level (Yes/No responses)** |
|  | Please kindly signify your organization’s adoption category |
| Category A | My organization currently uses robotics for its activities and executing its tasks |
| Category B | My organization plans to adopt robotics during the year |
| Category C | My organization is not using and has no plans to use robots in the nearest future |
| **Code** | **Survey Questions** |
| Q1 | Robotic technologies are easy for me to use in performing accounting tasks. |
| Q2 | Learning to use robots to perform accounting work is easy for me |
| Q3 | I have sufficient technical skills to operate robotic systems in accounting tasks. |
| Q4 | Applying robotics to my daily accounting tasks would be easy for me. |
| Q5 | I believe a robot would enable professional accountants to accomplish tasks more quickly |
| Q6 | Using a robot to perform task would save me a lot of time at work |
| Q7 | I believe that using robotics to perform daily tasks will reduce costs |
| Q8 | I believe using robots would improve my performance by reducing errors |
| Q9 | Robots will increase the value that I provide in my work |
| Q10 | Having robots would allow me to use my time to accomplish other tasks |
| Q11 | Using a robot will make my work easier and more efficient. |
| Q12 | I believe that robots will provide value when performing tasks |
| Q13 | I would trust the results provided from a robot when performing tasks |
| Q14 | I am interested in learning about robotics and how to apply them in accounting and auditing jobs. |
| Q15 | I would like to use robot for my work to gain more professional status |
| Q16 | I belief using robot will advance my career progression for promotions, opportunities, and remuneration |
| Q17 | I believe that robots will provide reliable results for tasks that are repetitive better than humans |
| Q18 | My organization supports the use of robots for professional accounting services |
| Q19 | My manager encourages and supports the use of robots in performing accounting work |
| Q20 | The professional bodies and auditing communities that I belong to supports the use of robotics |
| Q21 | My organization has established structures to support learning and using robotics |
| Q22 | The expectations of my clients and professional accounting bodies are that professional accountants should use robotics in performing routine work or audits |
| Q23 | The South Africa national government has policies and initiatives that encourage/support the adoption of RPA technologies by accounting professionals |
| Q24 | Other firms within the industry have adopted RPA, and there are industry-wide resources to support its use for accounting processes |
| Q25 | I intend to use robotics to perform daily accounting tasks in the future |
| Q26 | I predict that robots will be used in my organization for performing daily routine work in the near future |
| Q27 | I intend to learn how to use robotics soon |
